# Nodular Posterior Scleritis Mimicking Non-pigmented Choroidal Melanoma: A Case Report and Literature Review

**DOI:** 10.7759/cureus.73173

**Published:** 2024-11-06

**Authors:** Mariam N Alenezi, Salah Alrashidi, Ghassan Zein

**Affiliations:** 1 Ophthalmology, Farwaniya Hospital, Farwaniya, KWT; 2 Oncology and Retina Unit, Kuwait Sidra Hospital, Kuwait City, KWT; 3 Ophthalmology, Ahmadi Hospital, Ahmadi, KWT

**Keywords:** choroidal melanoma, mimicking lesions, nodular posterior scleritis, non-pigmented choroidal melanoma, posterior scleritis

## Abstract

Nodular posterior scleritis is an uncommon inflammatory disorder of the eye characterized by nodular lesions that may be similar to other intraocular conditions such as choroidal melanoma, leading to diagnostic difficulties. This case report and literature review aims to evaluate the demographics, modalities, treatment, and outcomes of nodular posterior scleritis.

We describe a 41-year-old female patient who presented with diminished vision in her left eye. Based on an examination and fundus findings, it was suspected to be non-pigmented choroidal melanoma. A multidisciplinary evaluation did not show evidence of an underlying systemic disease. The diagnosis was later confirmed to be nodular posterior scleritis based on imaging features and response to treatment. Previous reports of nodular posterior scleritis mimicking choroidal melanoma were viewed.

The patient was treated with systemic corticosteroids (prednisolone 40 mg daily), topical steroids, and mydriatic drops. Symptoms resolved and visual acuity improved in the interim, as demonstrated at the six-month follow-up.

This case underscores the significance of a comprehensive diagnostic workup to make an accurate diagnosis based on careful imaging and clinical evaluation that will aid in optimal management. Although MRI has largely replaced B-scan ultrasonography for imaging most intraocular masses, B-scan ultrasonography remains an important part of the initial assessment in cases of posterior scleritis to differentiate it from malignant masquerade syndromes and direct the appropriate therapeutic strategy.

## Introduction

Rarely, the presentation of posterior scleritis is through nodular lesions. In the absence of certain characteristic features, it is difficult to diagnose it with certainty as it may masquerade as other ocular pathologies, including choroidal tumors. Of these, non-pigmented choroidal melanoma can mimic nodular posterior scleritis to a great extent clinically and radiologically, leading to diagnostic challenges.

Choroidal melanoma is the most common primary malignant intraocular tumor in adults. The pigmented mass of choroidal melanoma is most often the presenting feature, but non-pigmented forms (amelanotic) also have been described and make uveal melanoma difficult to distinguish from other lesions on color alone [1].

Nodular posterior scleritis is a type of inflammation that affects the posterior segment of the sclera. Posterior scleritis is further divided into diffuse posterior scleritis and nodular posterior scleritis [2]. It is frequently associated with systemic diseases, such as rheumatoid arthritis, systemic lupus erythematosus, and other autoimmune conditions, while it can also happen idiopathically. Nodular posterior scleritis clinical features are vision loss, ocular pain, and headache, which are common findings even though it is difficult to distinguish with malignancies due to resemblant imaging characteristics [3].

The diagnosis of nodular posterior scleritis is often difficult, especially with respect to the differential diagnosis of choroidal melanoma. Imaging modalities containing ultrasonography, optical coherence tomography (OCT), and fluorescein angiography are essential in differentiating these conditions. Ultrasonography may identify a mass, with or without internal echoes, and OCT can reveal changes in the retinal and choroidal layers [4]. Unfortunately, the imaging findings of these studies may overlap and lead to diagnostic uncertainty. Histopathological examination is still considered the gold standard for the diagnosis of neoplasia and not posterior scleritis, but first, it is not done because of its invasive nature [5].

These reports bring to light the diagnostic dilemmas and therapeutic implications of posterior scleritis in simulating choroidal melanoma. A review by Shields et al. considered the different imaging modes and their implications in identifying whether the lesion of an eye is neoplastic or not [6]. Improved diagnostic imaging has led to more accurate diagnosis, but similar clinical presentations may require complete diagnostic workup with invasive procedures for definitive identification.

This case report aims to highlight the presentation of features constituting a clinical masquerade, which may suggest non-pigmented choroidal melanoma such as nodular posterior scleritis. This report aims to improve the differentiation between these two entities by describing the clinical presentation, imaging findings, and management strategy. A literature review will be presented to draw upon the broader framework of current knowledge and diagnostic practices in order to properly place this case.

## Case presentation

A 41-year-old woman visited the ophthalmology clinic at Farwaniya Hospital in Kuwait with complaints of reduced vision in her left eye. Her medical history included a previous stenting procedure for bile duct obstruction, but she had no history of eye trauma or prior surgeries.

On examination, her right eye had an uncorrected visual acuity (UCVA) of 20/25, with a clear cornea, normal conjunctiva, and a healthy anterior chamber. The intraocular pressure was within normal limits (18 mmHg), and extraocular muscle motility was full. The posterior segment showed no significant findings.

**Figure 1:**
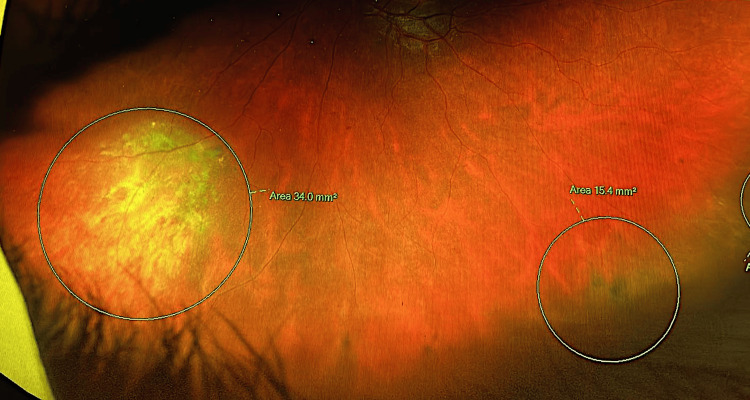
Wide field retinal imaging showing a mass in the lower inferonasal area

In her left eye, however, the UCVA was 20/40. The examination revealed similar clear anterior structures, but the fundus examination raised suspicion for non-pigmented choroidal melanoma due to a finding in the lower inferonasal area (Figure 1). This prompted further investigations.

**Figure 2:**
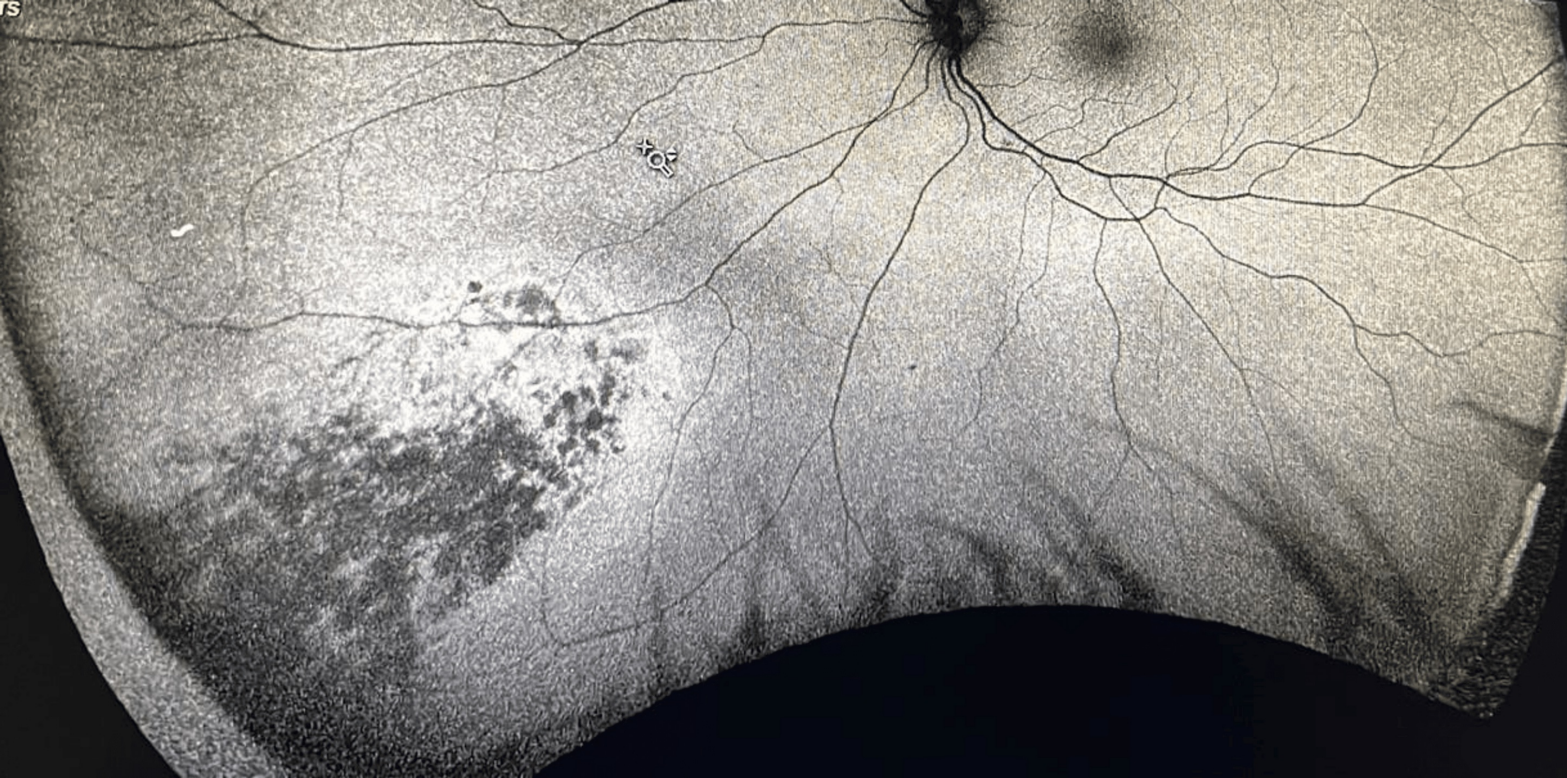
Fundus fluorescein angiography showing patchy hyperfluorescence in the lower inferonasal area corresponding to the suspected mass

**Figure 3:**
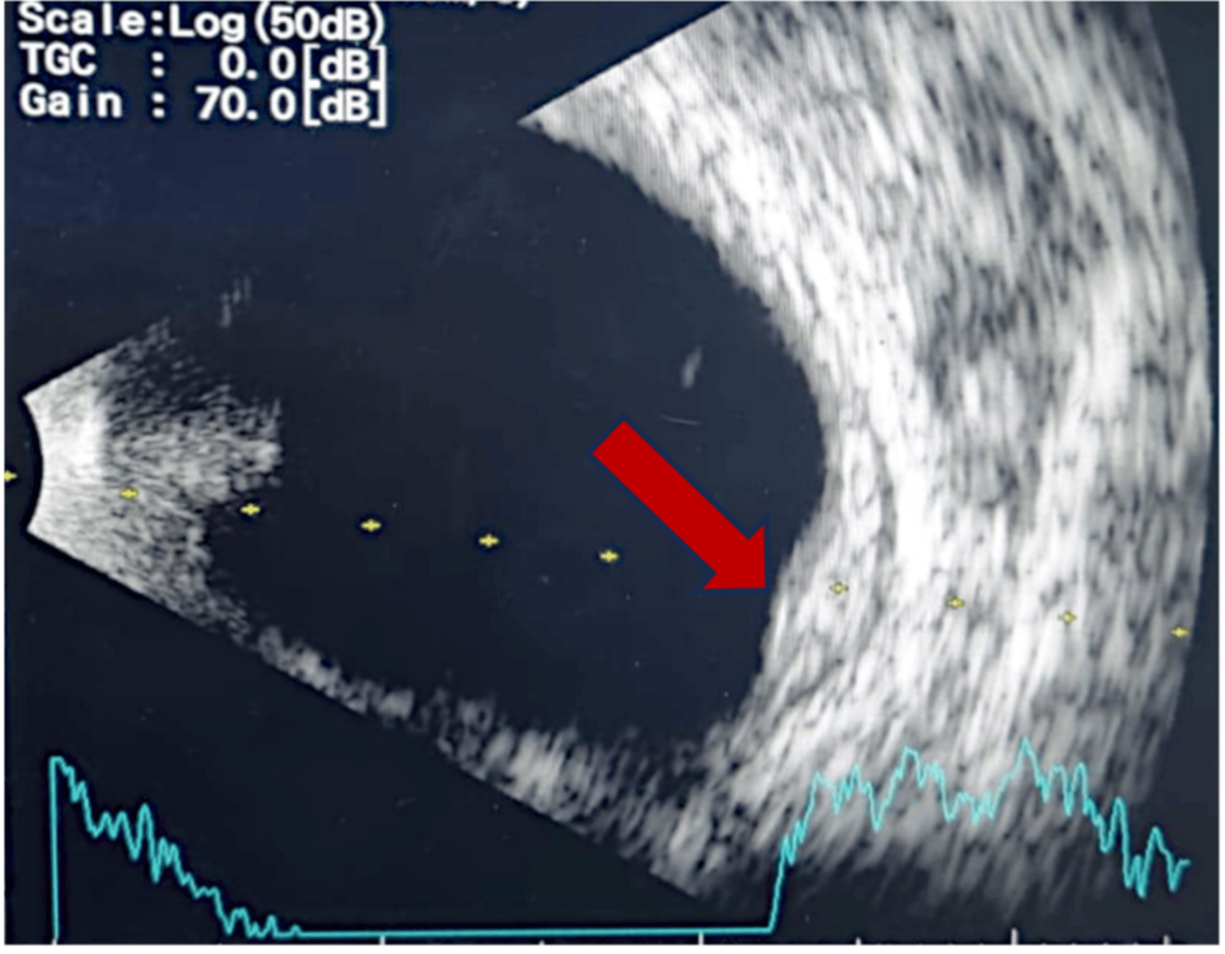
B-scan ultrasonography showing a thickened choroid with fluid in the subtenon's space (red arrow)

B-scan ultrasonography showed a thickened choroid with fluid in the subtenon's space, and fundus fluorescein angiography indicated patchy hyperfluorescence in the area corresponding to the suspected mass (Figures 2-3).

Both CT and MRI scans of the orbit excluded malignancy. Blood tests, including autoimmune and serology screenings, returned normal results. Eventually, we confirmed a diagnosis of left posterior scleritis based on these findings.

**Figure 4:**
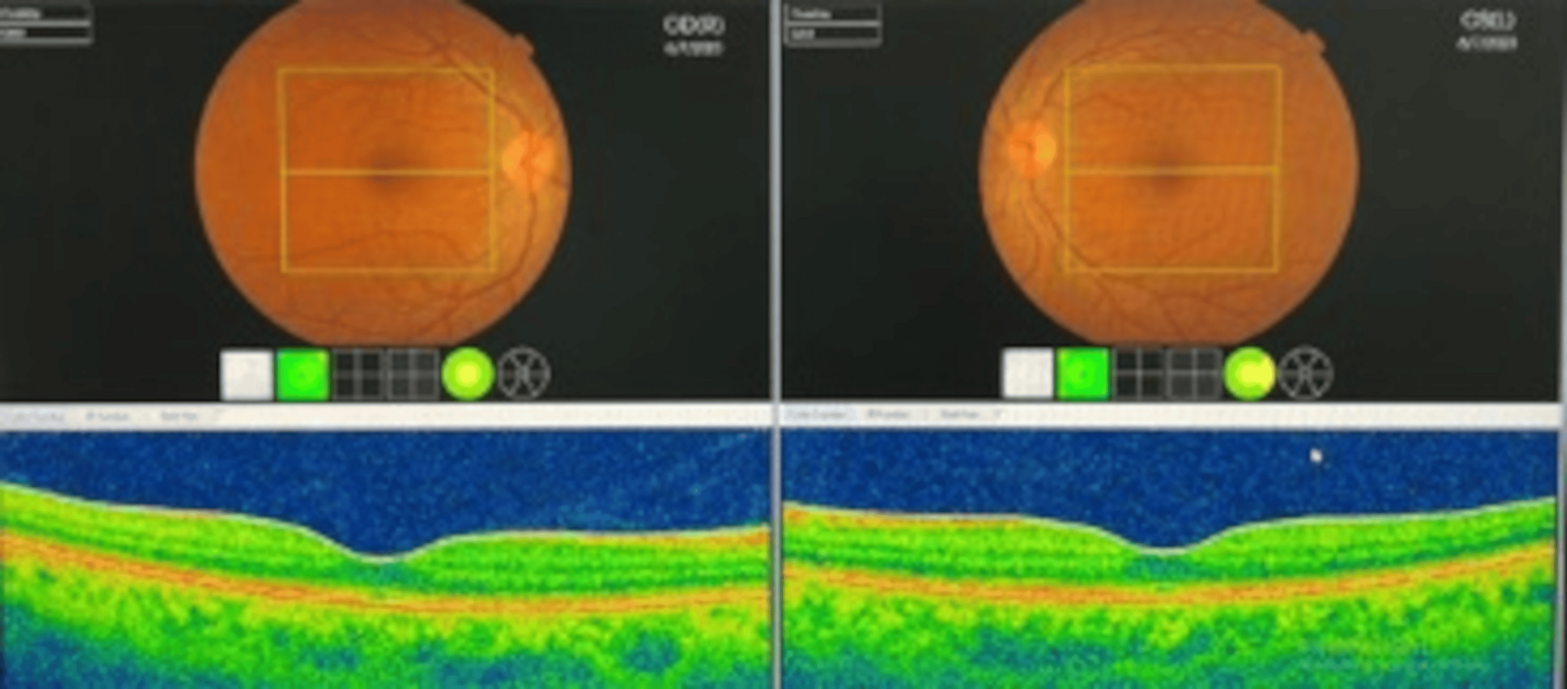
Bilateral optical coherence tomography six months after treatment

The patient was treated with systemic corticosteroids (prednisolone 40 mg daily), along with topical steroids and mydriatic eye drops. By her six-month follow-up, she had shown remarkable improvement (Figure 4).

## Discussion

Nodular posterior scleritis and non-pigmented choroidal melanoma present significant diagnostic challenges due to their overlapping features.

A total of 22 case reports were identified during the literature search that was done through PubMed and Google Scholar. Among the 22 case reports obtained, all involved patients with nodular posterior scleritis that mimicked amelanotic choroidal melanoma.

The mean age of the patients was 49.1 years, ranging from 30 to 72 years. Female patients made up 68% of the documented cases (15 cases), whereas males constituted 32% of cases (7 cases).

Initial visual acuity varied widely, from counting fingers to 20/20. Most patients treated with steroids experienced substantial improvement in vision, although one case, where vision remained at counting fingers, did not respond as well.

Treatment generally involved steroids administered as eye drops, orally, by injection, or intravenously. However, three cases were treated with non-steroidal anti-inflammatory drugs (NSAIDs). Patients receiving NSAIDs experienced relatively rapid and complete resolution of nodular posterior scleritis within two to four weeks, whereas those on corticosteroid therapy often took longer to recover.

Posterior scleritis is an inflammatory condition that requires ongoing follow-up for systemic inflammatory disorders. Of the 22 cases reported, only six (27.2%) were associated with an underlying autoimmune disease, while the remaining 16 cases (72.8%) were idiopathic.

The choice of imaging for diagnosing nodular posterior scleritis is critical. B-scan ultrasonography is often the most effective investigation, providing vital information about scleral thickening and fluid in the subtenon's space-key indicators of posterior scleritis [7]. In this case, it played a pivotal role in visualizing these features, aiding in the differentiation from non-pigmented choroidal melanoma. In contrast, treating choroidal melanoma involves various strategies, including plaque radiotherapy, proton beam therapy, or enucleation for advanced cases [8]. The literature review summarized in Table 1 highlights patients who were initially suspected of having choroidal melanoma but were ultimately diagnosed with nodular posterior scleritis.

**Table 1:** Basic characteristics of the included studies F: female, M: male, FA: fluorescein angiography, FFA: fluorescein fundus angiography, MRI: magnetic resonance imaging, OCT: optical coherence tomography, BCVA: best corrected visual acuity, CF: counting fingers, ICGA: indocyanine green angiography, IV: intravenous, NSAID: non-steroidal anti-inflammatory drugs, CT: computed tomography, NIL: nothing, SLE: systemic lupus erythematosus

Author	Year	Initial diagnosis	Diagnosis	Gender	Age	Eye affected	Investigation	Associated autoimmune illness	Vision before treatment	Vision after treatment	Country	Treatment	Outcome
Dubey et al. [9]	2024	Choroidal melanoma	Nodular posterior scleritis	F	38	Right	Ultrasonography, FA	No autoimmune, has a history of choroidal melanoma in her left eye	20/40	20/20	India	3 cycles of IV methylprednisolone and oral corticosteroids	Resolution of mass size and improvement of vision 20/20 after 6 weeks
Babu et al. [10]	2021	Amelanotic choroidal melanoma	Nodular posterior scleritis	M	57	Right	MRI, FFA, OCT	NIL	BCVA 20/1200	BCVA 20/60	India	1 mg/kg oral steroids	Complete regression of the mass lesion after 3 months
Khadka et al. [11]	2021	Choroidal melanoma	Nodular posterior scleritis	M	52	Left	B-scan, OCT, FFA, ICGA, MRI	NIL	BCVA 6/12	BCVA 6/6	Nepal	1 gram intravenous methylprednisolone for 3 days, then oral prednisolone 60 mg daily tapered over 6 weeks	Complete regression of choroidal lesion after 1 year follow-up
Maksimova [12]	2021	Amelanotic choroidal melanoma	Atypical posterior scleritis	F	54	Left	B-mode ultrasound, OCT, MRI	NIL	BCVA 10/20	__	Spain	Oral prednisone 60 mg/day for 20 days	Significant improvement
Awh et al. [13]	2020	Amelanotic choroidal lesion	Nodular posterior scleritis	M	67	Left	B-scan ultrasonography, FA, OCT, CT, MRI	Giant cell arteritis	BCVA 20/70	CF	United States of America	High dose oral prednisone 60 mg daily	Vision remained CF
Alsharif and Al-Dahmash [14]	2018	Amelanotic choroidal melanoma	Atypical idiopathic non-necrotizing posterior scleritis	M	30	Left	B-scan, MRI	NIL	Visual acuity 20/200	Visual acuity 20/28.5	Saudi Arabia	Prednisolone 1 mg/kg for 2 weeks, then transseptal periocular triamcinolone acetonide (40 mg/ml)	Significant reduction of choroidal mass, and improvement of vision after 1 month after injection
Sin [15]	2016	Choroidal tumor	Nodular posterior scleritis	F	42	Right	B-scan, FA, OCT, MRI	SLE	20/60	20/30	China	Topical steroid treatment, IV methylprednisolone 1 g daily for 3 days, Discharged with oral prednisolone, 20 mg per day for 2 weeks	complete resolution of the choroidal mass after discharge in 2 weeks. However, a new lesion of 1 disc diameter in size and extrafoveal serous chorioretinopathy had emerged in her left eye that then resolved, VA was stable at 20/30
Liu et al. [16]	2015	Amelanotic choroidal melanoma	Nodular posterior scleritis	F	42	Left	B-mode ultrasound, FA, ICGA, MRI	NIL	BCVA 20/40	BCVA 20/20	China	Oral NSAID (indomethacin 25 mg TDS)	Complete resolution of choroidal mass after 2 months of NSAID treatment.
Ozkaya et al. [17]	2015	Choroidal mass	Nodular posterior scleritis	F	41	Right	B-scan, OCT, FA, ICGA	NIL	BCVA 20/50	BCVA 20/20	Saudi Arabia	Nepafenac eye drops 3 times a day, and flurbiprofen tablet 100 mg twice a day for 4 weeks	Complete regression of subretinal mass and decrease in subretinal fluid
Hage et al. [18]	2011	Choroidal metastases	Nodular posterior scleritis	F	38	Right	FA	Isolated inflammatory syndrome	BCVA 20/80	BCVA 20/20	France	Corticosteroid drops with mydriatics, oral corticotherapy	Fundus exam was normal after 3 weeks
Hage et al. [18]	2011	Choroidal metastasis	Nodular posterior scleritis	F	52	Right	FA, OCT, MRI	NIL	20/200	__	France	NSAID eye drops	Improvement of visual acuity, normal fundus after 2 weeks FA showed retinal pigment epithelium atrophy
Hatef et al. [19]	2010	Choroidal malignancy	Nodular posterior scleritis	M	55	Right	Ocular ultrasonography	NIL	20/40	20/20	USA	IV methylprednisolone 1 g daily for 3 days then oral prednisolone 60mg daily+ mycophenolate mofetil 2500 mg/d	Recovery after 11 months
Wang et al. [20]	2010	Choroidal mass	Nodular posterior scleritis	F	72	Right	FA, OCT, B-scan ultrasonography	SLE, alopecia	20/32	20/32	Singapore	Topical, multiple periocular steroid injections	Complete recovery
Shukla [21]	2006	Choroidal melanoma	Nodular posterior scleritis	F	45	Right	B-scan ultrasonography, FA	NIL	BCVA 17/200	BCVA 20/20	__	IV methylprednisolone 15 mg/kg/day for 3 days then 50 mg/days	Complete resolution with visual improvement after 1 month
Wong et al. [22]	2003	subretinal mass	Posterior scleritis	F	40	Left	A-scan ultrasonography	NIL	BCVA 20/30	BCVA 20/20	Taiwan	Oral prednisolone 40 mg + NSAID	Resolution after 3 weeks
Arevalo et al. [23]	2003	Choroidal melanoma	Nodular posterior scleritis	F	37	Left	FA, ICGA, ultrasonography	NIL	20/60	__	United States of America	Oral steroids	No change after 3 weeks
Osman Saatci et al. [24]	2000	Choroidal tumor mass	Posterior scleritis	M	36	Right	Ultrasonography, MRI	NIL	CF	__	Turkey	Oral 80 mg daily prednisolone for 2 weeks, then tapered down	Complete resolution at 6 months and improvement of visual acuity
Demirci et al. [25]	2000	Choroidal melanoma	Nodular posterior scleritis	F	41	Left	FA, A- and B-scan ultrasonography, MRI, scleral biopsy	NIL	20/20	20/20	United States of America	Observation	Unchanged fundus lesion
Gedde and Augsburger [26]	1994	Fundal mass	Posterior scleritis	F	62	Left	__	NIL	20/70	20/20	United States of America	Oral prednisolone 80 mg/d for 1 week	Reduction in size after 1 week and resolution after 3 months
Gupta et al. [27]	1992	Choroidal melanoma	Nodular posterior scleritis	F	45	Left	FA, OCT, B-scan ultrasonography	Rheumatoid arthritis	20/60	__	India	Oral prednisolone (2mg/kg/d) for 10 days then tapered over 3 months, maintained on low dosage cyclophosphamide for 1 year	Mass resolution in 10 days
Finger et al. [28]	1990	Choroidal melanoma	Posterior scleritis after pathology findings	M	66	Left	A-scan ultrasonography, FA, B-scan	Transient episode of polyarthritis	BCVA 20/200	__	United States of America	Enucleated and received 600 cGy of preoperative external beam radiotherapy	Outcome not specified
Brod and Saul [29]	1990	Choroidal melanoma	Nodular posterior scleritis	F	70	Left	Ultrasonography, FA	NIL	20/200	20/30	United States of America	Systemic corticosteroids	Improvement after 4 weeks

This case underscores the need for a thorough diagnostic evaluation when encountering ocular lesions that can easily be mistaken for one another. While corticosteroids effectively treat posterior scleritis, non-pigmented choroidal melanoma requires entirely different therapeutic approaches tailored to the tumor's characteristics. Accurate imaging and clinical assessment are essential in guiding both diagnosis and management.

## Conclusions

In this case report, we present an example of how nodular posterior scleritis can clinically masquerade as non-pigmented choroidal melanoma with a detailed description of diagnostic and treatment challenges. The patient presented with malignancy the first time, but imaging and clinical evaluation were made to diagnose posterior scleritis.

The diagnosis was confirmed by B-scan ultrasonography showing blood in the outer layer of the scleral thickening with fluid therein, thus ruling out malignancy. The treatment was largely limited to systemic corticosteroids, which immediately improved the symptoms, but NSAIDs worked poorly as monotherapy. On the opposite side, non-pigmented choroidal melanoma should be managed with a different approach than that of plaque radiotherapy or enucleation. However, the accurate distinction of these entities is instrumental for both therapeutic actions and ultimately patient well-being.

This case report highlights the need for a comprehensive diagnostic workup including detailed imaging and clinical evaluation to differentiate nodular posterior scleritis from choroidal melanoma in order to increase optimal patient management through appropriate therapy strategies.
